# Resistance to bond degradation between dual-cure resin cements and pre-treated sintered CAD-CAM dental ceramics

**DOI:** 10.4317/medoral.17828

**Published:** 2012-02-09

**Authors:** Raquel Osorio, Raquel Castillo-de Oyagüe, Francesca Monticelli, Estrella Osorio, Manuel Toledano

**Affiliations:** 1 Professor. Department of Dental Materials, School of Dentistry. University of Granada (UGR), Granada, Spain; 2 Associate Professor. Department of Buccofacial Prostheses, Faculty of Odontology. Complutense University of Madrid (UCM), Madrid, Spain; 3 Tenured Professor. Department of Surgery, Faculty of Sport and Health Sciences. University of Zaragoza, Campus de Huesca, Huesca, Spain

## Abstract

Objective: To evaluate the bond stability of resin cements when luted to glass-reinforced alumina and zirconia CAD/CAM dental ceramics. Study design: Eighteen glass-infiltrated alumina and eighteen densely sintered zirconia blocks were randomly conditioned as follows: Group 1: No treatment; Group 2: Sandblasting (125 µm Al2O3-particles); and Group 3: Silica-coating (50 µm silica-modified Al2O3-particles). Composite samples were randomly bonded to the pre-treated ceramic surfaces using different resin cements: Subgroup 1: Clearfil Esthetic Cement (CEC); Subgroup 2: RelyX Unicem (RXU); and Subgroup 3: Calibra (CAL). After 24 h, bonded specimens were cut into 1 ± 0.1 mm2 sticks. One-half of the beams were tested for microtensile bond strength (MTBS). The remaining one-half was immersed in 10 % NaOCl aqueous solution (NaOClaq) for 5 h before testing. The fracture pattern and morphology of the debonded surfaces were assessed with a field emission gun scanning electron microscope (FEG-SEM). A multiple ANOVA was conducted to analyze the contributions of ceramic composition, surface treatment, resin cement type, and chemical challenging to MTBS. The Tukey test was run for multiple comparisons (p < 0.05). Results: After 24 h, CEC luted to pre-treated zirconia achieved the highest MTBS. Using RXU, alumina and zirconia registered comparable MTBS. CAL failed prematurely, except when luted to sandblasted zirconia. After NaOClaq storage, CEC significantly lowered MTBS when luted to zirconia or alumina. RXU decreased MTBS only when bonded to silica-coated alumina. CAL recorded 100 % of pre-testing failures. Micromorphological alterations were evident after NaOClaq immersion. Conclusions: Resin-ceramic interfacial longevity depended on cement selection rather than on surface pre-treatments. The MDP-containing and the self-adhesive resin cements were both suitable for luting CAD/CAM ceramics. Despite both cements being prone to degradation, RXU luted to zirconia or untreated or sandblasted alumina showed the most stable interfaces. CAL experimented spontaneous debonding in all tested groups.

** Key words:**CAD/CAM ceramic, alumina, zirconia, resin cement, surface pre-treatment, sandblasting, silica-coating, chemical aging, bond degradation, microtensile bond strength.

## Introduction

Densely sintered zirconia and glass-infiltrated alumina have expanded clinical indications for all-ceramic tooth- and implant-supported fixed dental prostheses (FDPs) (1,2).

Even when CAD/CAM-based ceramic restorations can be fixed with conventional cements such as zinc-phosphate or resin-modified glass-ionomer, adhesive cementation has been recommended for improving the clinical retention and marginal fit (1).

Nevertheless, searching for a suitable luting strategy to achieve durable bonds between resin cements and high-strength ceramic cores is still a matter of concern (3,4). Alternative porcelain surface treatments such as sandblasting or silica-coating have been suggested, as neither hydrofluoric acid etching nor silanization result in a satisfactory bond to alumina or zirconia due to the absence of a silicon oxide phase in their composition (4,5). However, the influence of conditioning methods on resin bond strength and durability has not been accurately quantified for CAD/CAM dental ceramics (6,7).

New dual-cure resin cements, such as the phosphate monomer-containing Clearfil Esthetic Cement and the self-adhesive RelyX Unicem have widely been indicated for luting sintered ceramic cores (4,6,8). However, little information is available in the literature about the longe-vity of these bonds (8,9); and many factors, e.g., ceramic wettability, porcelain surface roughness or the bonding agents’ composition and performance may affect the quality of the resin cement/ceramic adhesion (10).

Furthermore, acidic compounds in dentinal fluids, salivary enzymes, and proteolytic residues produced by oral bacteria may hamper the stability of adhesive interfaces (10,11), and have recently been considered as potential sources of chemical bond degradation (10,12). Besides, bonded ceramic restorations promote higher crevicular fluid accumulation (with bacterial products and host-derived factors) than do dental tissues (13,14).

Based on the presence of sodium azide in artificial saliva, immersion in a sodium hypochlorite aqueous solution (NaOClaq) has been proposed as a suitable and less time-consuming aging technique (15,16) that may reproduce in just a few hours the long-term hydrolytic effect of the mentioned bond biodegradators present in saliva (12,17-19). Thus, NaOCl solutions have been described as potent deproteinizing and biological oxidants with the capability of accelerating natural bond deterioration (18). Such storage medium has been considered to have similar efficacy and lower variability than water aging or thermocycling (16).

Because the aim of this study was to test the resistance to chemical degradation of different dual-cure resin cements luted to pretreated alumina and zirconia ceramic surfaces, immersion of bonded specimens in NaOClaq was performed. The null hypothesis tested was that neither ceramic composition, nor conditioning method, nor resin cement type influence the bond stability of resin cement/ceramic interfaces.

## Material and Methods

-Experimental design

Eighteen cubic-shaped (edge = 19.5 mm) sintered and glass-infiltrated (15 vol % quartz glass) blocks of alumina (batch no. 7803, Al Cubes for Cerec, Vita Zahnfabrik; Bad Säckingen, Germany) and eighteen cylinder-shaped (Ø19.5 mm × 5.25 mm high) sintered zirconia blanks (batch no. 18004627, Cercon Zirconia, Dentsply; Konstanz, Germany) were selected for the study. CAD-CAM aluminum and zirconium oxide ceramic blocks were randomly divided into three groups (n = 6 for each ceramic type) according to the different pre-treatments carried out: Group 1: No surface treatment; Group 2: Sandblasting using 125 μm alumi-num-oxide (Al2O3) powder (Supradental; Madrid, Spain) applied perpendicularly to the ceramic surface for 10 s at a working distance of 5 mm under a pressure of 75 ± 10 psi; and Group 3: Tribochemical silica-coating with 50 μm Al2O3-particles modified by silica oxide (Supradental; Madrid, Spain). Thirty-six composite specimens (height = 4 mm) were made by layering 2 mm-thick increments of a microhybrid composite (batch no. J27435, Tetric Evo Ceram, Ivoclar-Vivadent; Schäan, Liechtenstein) using a square silicon mold for the alumina cubes and a rounded one for the zirconia ceramic cylinders. Each composite film was condensed with a clean plastic filling instrument to avoid contamination, and light-cured for 40 s at 600 mmW/ cm2 (BluePhase, Ivoclar-Vivadent; Schäan, Liechtenstein). The last increment was compressed using a glass microscope slide in order to obtain a flat surface. After removing the specimens from the mold, the portions that were previously in contact with the silicone pattern were irradiated for an extra 40 s.

-Luting procedure

Composite samples were randomly bonded to the treated ceramic surfaces using different resin cements: Subgroup 1 (CEC) used an MDP-containing resin cement: Clearfil Esthetic Cement (batch no. 00002A/0001AB, Kuraray Medical; Okayama, Japan); Subgroup 2 (RXU) used a self-adhesive resin agent: RelyX Unicem (batch no. 245776, 3M ESPE; Seefeld, Germany); and Sub-group 3 (CAL) used a conventional Bis-GMA resin cement: Calibra (batch no. 060112 (base) / 051151 (catalyst), DeTrey Dents-ply; Konstanz, Germany). All materials were handled following the manufacturer’s instructions at room temperature (RT) of 23.0°C ± 1.0°C. The chemical composition and application mode of the cements tested are detailed in  Table 1.

**Table 1:**
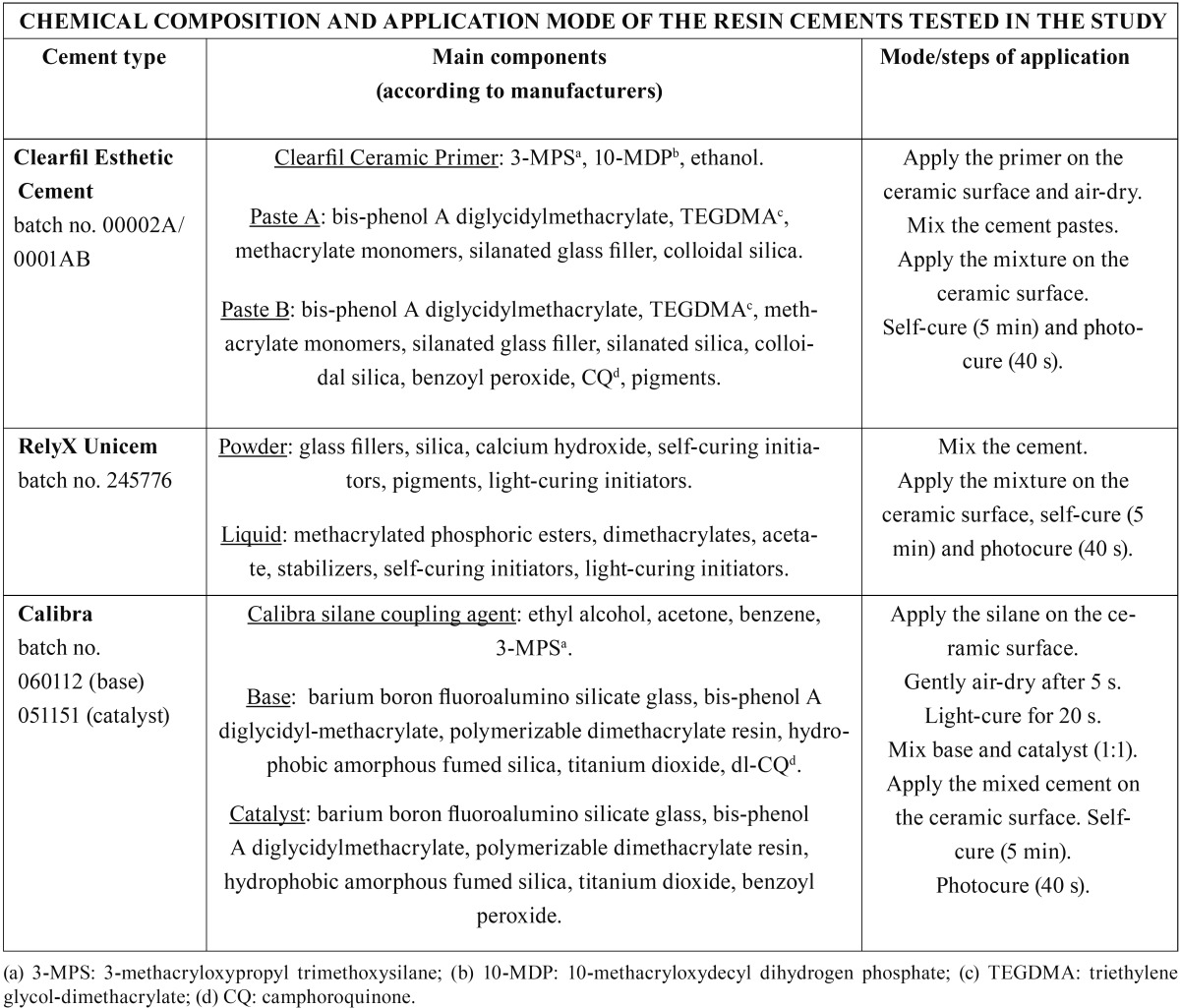
Chemical composition and application mode of the resin cements tested in the study.

The ceramic-to-composite luting procedures were carried out by means of a customized metallic tool that produced a constant seating pressure of 1 kg (1,249 MPa). The compressive force was applied for 5 min, leaving the resin cement to set in the self-curing modality. Finally, the specimens were photo-activated for 40 s on each side of the blocks (BluePhase: 600 mmW/ cm2) to ensure optimal polymerization. Bonded specimens were stored in distilled water for 24 h at 37 °C.

-Microtensile bond strength test

After 24 h of water storage, all samples were vertically sectioned under water cooling into 1 ± 0.1 mm2 sticks using a slow-speed diamond saw (Accutom 50, Struers GmbH; Copenhagen, Denmark) according to the “non-trimming” method of the microtensile test. One-half of the specimens from each experimental subgroup were tested for microtensile bond strength (MTBS). The re-maining one-half were stored in a 10 % sodium hypochlorite aqueous solution (NaOClaq) for 5 h. Beams were then retrieved from the challenging medium and tested in microtension.

Twenty microtensile sticks were obtained per subgroup. Each stick was attached with cyanoacrilate adhesive (Zapit, Dental Ventures of America; Corona, CA, USA) to the flat grip of a Bencor Multi-T testing assembly (Danville Engineering; San Ramon, CA, USA) and loaded in tension with a bench-top universal testing machine (Instron Model 4411, Instron; Canton, MA, USA) at a crosshead speed of 0.5 mm/min until failure. The detached area of the ceramic beams was measured with a pair of digital callipers. Microtensile bond strength (MTBS) values were calculated in MPa.

Failure modes were evaluated by a single operator under an optical microscope (BH-2 Olympus; Tokyo, Japan) at 70× magnifications, and classified as adhesive (at the cement/ceramic interface), cohesive (within the resin cement), or mixed (with both adhesive and cohesive phases).

-Statistical analysis

Normal data distribution was confirmed by the Kolmogorov-Smirnov test, and homogeneity of variances was verified according to the Levene’s test. A multiple ANOVA was conducted to analyze the contributions of ceramic composition, surface treatment, resin cement type, chemical challenging, as well as the interaction of these factors to MTBS. The Tukey’s test was run to make post-hoc multiple comparisons. Pre-test failures of the beams, that occurred spontaneously prior to microtensile testing, were counted as “zero bonds” (MPa = 0). The statistical significance was set at α = 0.05. All data analyses were made with SPSS/PC+ v.17.0 statistical software (SPSS Inc.; Chicago, IL, USA).

-Field emission gun scanning electron microscope (FEG-SEM) evaluation

Four representative sticks from each subgroup were rinsed with 96 % ethanol, mounted on metallic stubs, gold sputtered (Emitech k550×, Emitech; Ashford, UK), and evaluated by a single operator under a field emission gun scanning electron microscope (FEG-SEM: JSM-6330 F, Jeol; Tokyo, Japan) at different magnifications (from 95× to 1000×) using an accelerating voltage of 10 kV, in order to assess the fracture pattern and the morphology of the debonded surfaces.

## Results

Ceramic composition, surface treatment, resin cement type, and NaOClaq immersion significantly affected bond strengths to CAD-CAM ceramics (p < 0.001). Interactions were also significant except the interaction between ceramic composition and chal-lenging procedure (p = 0.558). The mean MTBS values (MPa) and the results of the post-hoc comparisons are outlined in  Table 2.

**Table 2:**
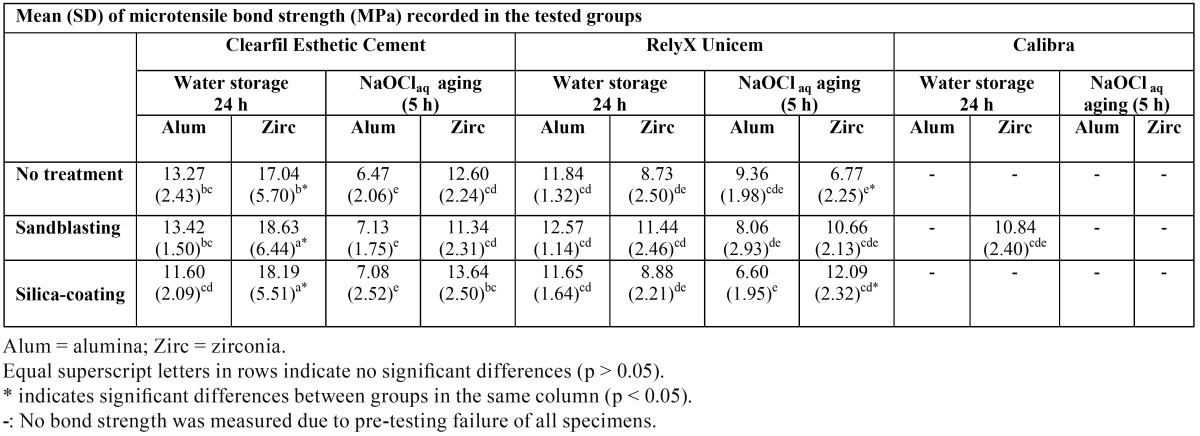
Mean (standard deviation) of microtensile bond strength (MPa) recorded in the tested groups.

After 24 h of water immersion, CEC bonded to pre-treated zirconia achieved the highest MTBS. CEC luted to zirconia showed significantly higher MTBS than that of RXU bonded to alumina or zirconia regardless of the surface treatment. Despite the conditioning method, both ceramics registered comparable MTBS when RXU was used; while CEC and RXU attained comparable results when luted to alumina. CAL samples failed prematurely excluding air-abraded zirconia surfaces, which showed comparable MTBS values to those of CEC luted to alumina, and RXU bonded to alumina or zirconia.

After NaOClaq storage, CEC significantly decreased MTBS in all subgroups. RXU lowered MTBS only when luted to silica-coated alumina. After challenging, CEC and RXU resulted in comparable MTBS when applied to sandblasted or silica-coated zirconia; whereas untreated zirconia bonded to CEC resulted in higher MTBS than when luted to RXU. CEC and RXU bonded to alumina showed similar MTBS values after aging. Adhesion of sandblasted zirconia surfaces luted with CAL fell after NaOClaq immersion, so that CAL recorded 100 % of pre-testing failures.

 Table 3 summarizes the failure mode distribution. At 24 h, the main failure type was mixed in all tested groups excepting zirconia beams luted with RXU, which mostly failed adhesively. Low percentages of cohesive fractures were only detected when CEC was bonded to untreated zirconia at 24 h. A complete detachment of CAL from the porcelain surface frequently occurred irrespective of the conditioning method. After challenging, adhesive failures augmented in all groups, with the exception of silica-coated zirconia sticks luted with RXU.

**Table 3:**
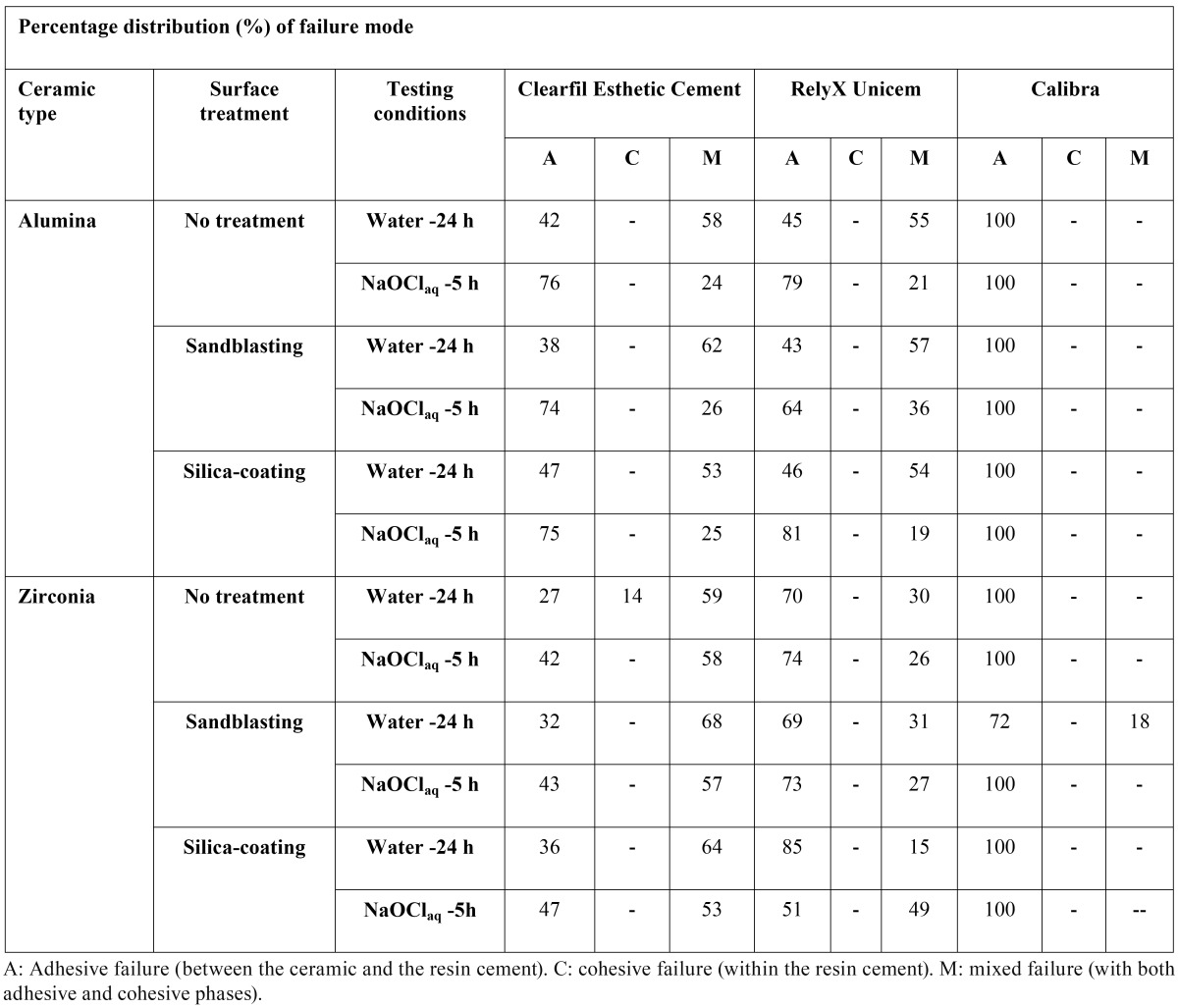
Percentage distribution (%) of failure mode registered in the experimental groups.

**Figure 1:**
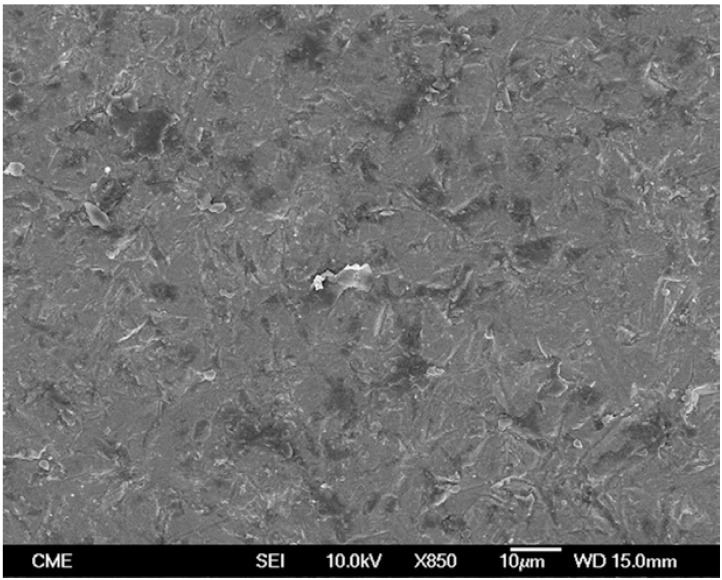
FEG-SEM micrographs of pre-treated ceramics. A) Sandblasted alumina surfaces exhibited edge-shaped micro-roughness (850×; bar 10 µm).

**Figure 2:**
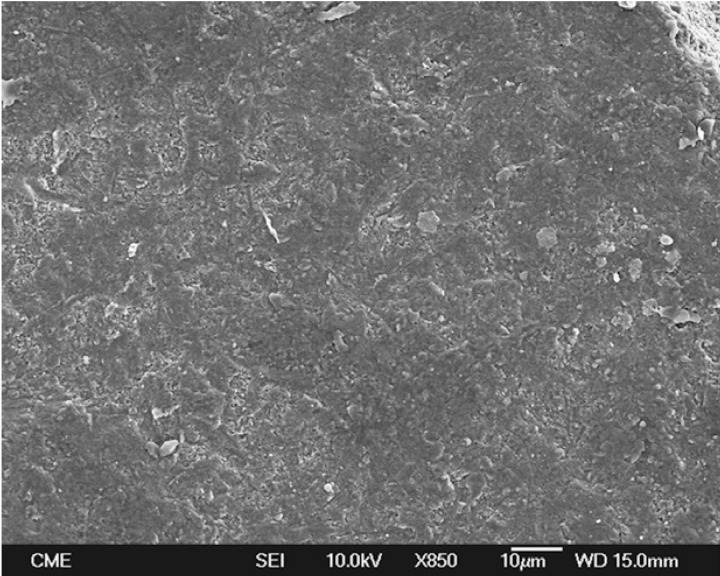
A slight undulation in the zirconia surface texture was observed after silica-coating (850×; bar 10 µm).

**Figure 3:**
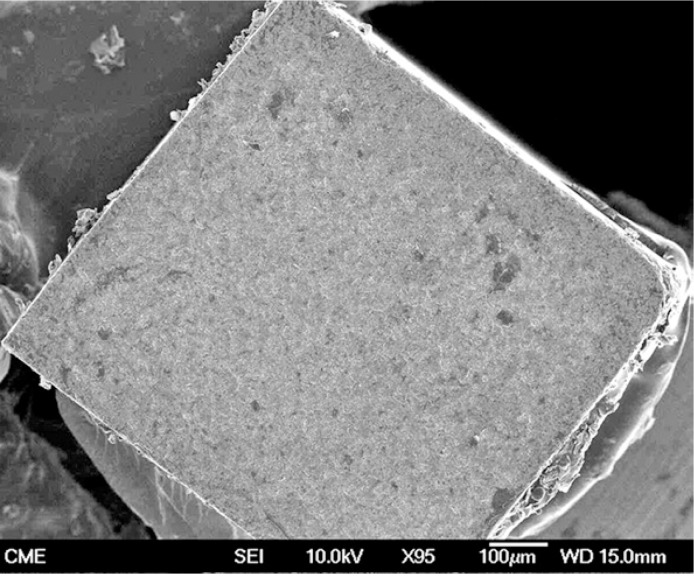
FEG-SEM figures of Clearfil bonded to a sandblasted alumina surface at 24 h. A) Cohesive failure: the entire porcelain surface was covered by a resin cement film (95×; bar 100 µm).

**Figure 4:**
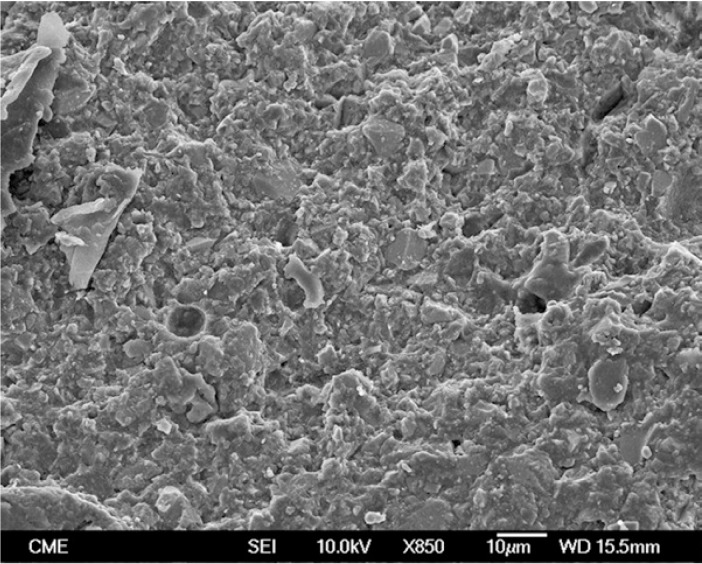
Sparse, scattered cement porosities were detectable at higher magnification (850×; bar 10 µm).

**Figure 5:**
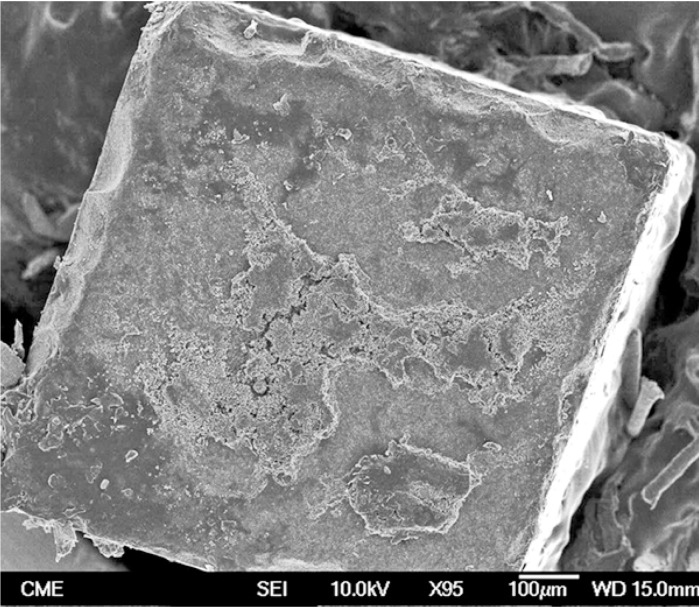
FEG-SEM images of fractured beams after NaOClaq storage. A) Mixed failure of a sandblasted alumina stick bonded with Clearfil, showing cement layers with protruding filler particles (95×; bar 100 µm).

**Figure 6:**
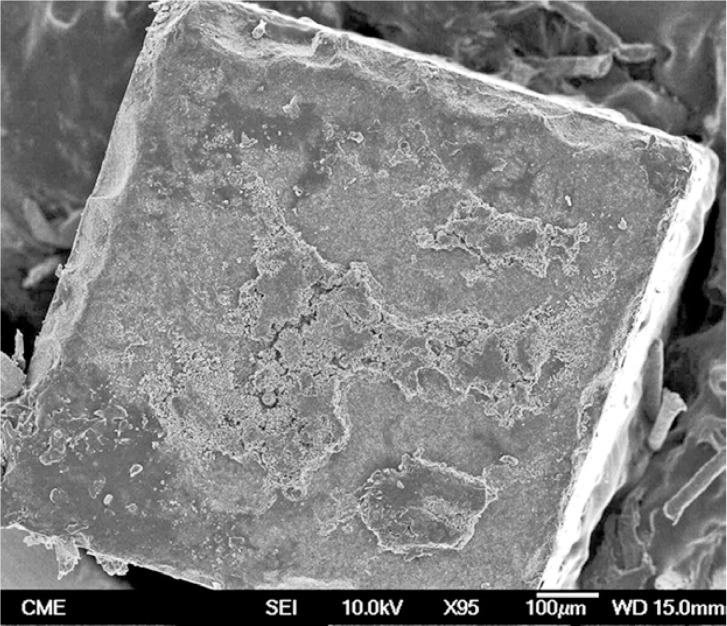
Micro-irregularities and dissolved cement residuals were noticed at higher magnification (850×; bar 10 µm).

**Figure 7:**
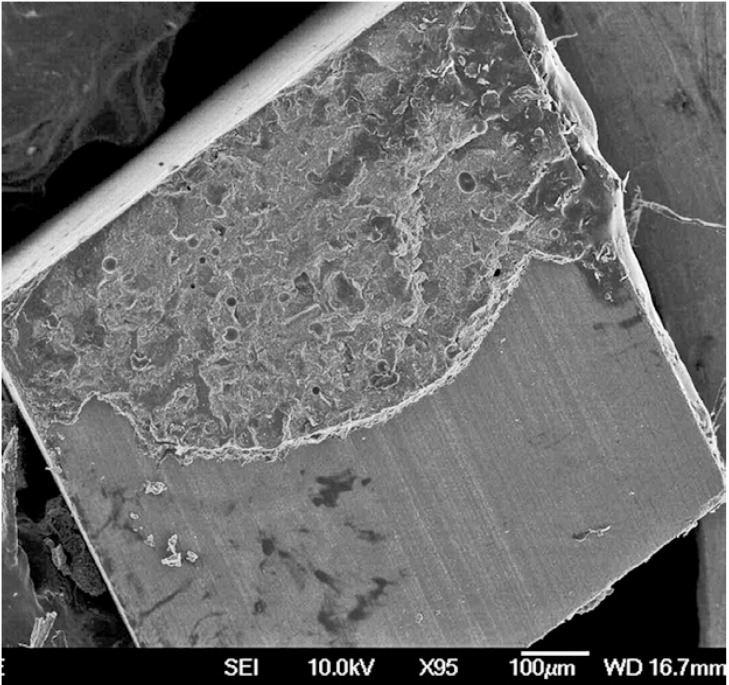
Mixed failure of an untreated zirconia surface luted with RelyX Unicem (95×; bar 100 µm). Cement dissolution areas with round-shaped margins remained at the top side of the beam (cohesive phase).

Representative FEG-SEM images are presented in figures 1 to 3. Sandblasted substrates evidenced edge-shaped micro-roughness (Fig. 1) whereas silica-coated beams showed a slight undulation in their surface texture with homogeneously distributed micro-irregularities (Fig. 2). At 24 h, remaining cement layers mainly luted to pretreated ceramics were observed (Figs. 3,4). After NaOClaq immersion, cement residuals of CEC (Figs. 5,6) and RXU (Fig. 7) persisted above the ceramic substrates.

## Discussion

Resin cement/ceramic bonded interfaces are susceptible to degradation. Challenging these bonding sites at the laboratory is required to test the bond stability of different luting systems proposed for oxide-based ceramics (1). A microtensile test was performed, since it allows for a homogeneous distribution of stress across the adhesive interface and a sensitive evaluation of bond strength (20). The findings of the present study require rejection of the null hypothesis because ceramic composition, conditioning treatment and resin cement type influenced the bond stability at the resin cement/ceramic interfaces.

At 24 h evaluation, the bond strength of CEC to pretreated zirconia reached the highest MTBS values ( Table 2). Prior to CEC application, the ceramic primer was spread over the porcelain surface to improve adhesion and to protect against moisture. 3-MPS (3-methacryloxypropyl trimethoxysilane) and 10-MDP (10-methacryloxy-decyl-dihydrogen phosphate) monomers present in the CEC ceramic primer composition (Table 1) optimize the surface wettability (21) and create cross-links: (a) with the 10-MDPs dispersed in the CEC resin matrix and (b) with the hydroxyl radicals (OH-) of the zirconia surfaces (2,22). Consistently, the main failure type recorded in the CEC groups before challenging was mixed ( Table 3).

In the first minute of the mixture of RXU, the pH is purported to be low, less than 2 pH units, due to the presence of multifunctional phosphoric methacrylates ( Table 1). Even though this acidity seemed to promote adhesion to a glass-porcelain (IPS Empress, Ivoclar Vivadent) in a previous study (23), it remains to be ascertained whether this chemical interaction also occurs when bonded to CAD/CAM ceramics (8). In fact, RXU mostly failed adhesively at 24 h ( Table 3).

CAL confirmed pre-testing failures with complete detachment from the ceramic substrates except when luting sandblasted zirco-nia surfaces ( Table 2, Table 3) due to their rough texture (4,8). An AFM analysis developed in a former investigation revealed a significant increase in the average roughness of zirconia surfaces after sandblasting (4). The lack of chemical interaction between CAL and the tested ceramics may be due to the absence of adhesive functional monomers in the composition of the resin cement and the silane coupling agent (24). Actually, the high content of solvents in the silane formulation may interfere with the resin polymerization, jeopardizing adhesion.

Concerning the ceramic type, glass-reinforced alumina has been reported to be rather resistant to air-abrasion and/or chemical surface reactions (25). This may explain why CEC and RXU attained comparable MTBS results despite the alumina surface pretreatment ( Table 2).

Several in vitro methods have been proposed to replicate the clinical conditions that may cause the adhesive interfaces to fail (15). Short-term immersion in 10 % NaOClaq has previously been used to reproduce the long-term action of salivary enzymes (10,18,19,26). In the current research this solution hampered the bond strength of CEC and CAL when combined with either type of ceramic and conditioning method.

After NaOClaq storage, the structural deterioration of CEC was observable such that the resin portion seemed to be dissolved displaying protruding filler particles (Figs. 5,6). The use of resin cements containing adhesive phosphate monomers has been deemed the best luting strategy for both alumina (27) and zirconia (28) mainly after air-abrading the ceramic surfaces. Nevertheless, microstructural changes in the CEC resin cement were noticeable in the comparison of FEG-SEM images taken before and after chemical aging (Figs. 4,6 respectively).

Sandblasting is also supposed to somewhat compensate for the lack of adhesive monomers in the CAL Bis-GMA matrix (4,8), but it has not contributed to bond stability ( Table 2). Moreover, the 3-MPS hydrophilic monomers of the silanes used prior to CEC and CAL resin cements may have expedited interfacial sorption and hydrolytic effects thanks to the establishment of hydrogen bonds throughout the hydrophilic groups (29). This could justify the bond strength decrease of both cements after challenging.

In RXU debonded specimens, cement dissolution areas (with rounded margins) were detected by FEG-SEM after chemical aging (Fig. 7). The adhesive interface of RXU has been compared to that of some conventional luting agents such as silicate or zinc-phosphate cements (30). The inorganic fillers (glass silicate) may react with the acidic phosphoric ester forming a silicate gel in which glass particles are entrapped. Therefore, RXU luted to zirconia and untreated or sandblasted alumina demonstrated greater bond stability than that of the CEC and CAL subgroups. Possible differences in effective silica deposition and nanomor-phological alterations produced by silica-coating on alumina and zirconia substrates deserve further investigation. Despite not being the focus of the study, it might contribute to explain why RXU decreased MTBS only when luted to silica-coated alumina. Overall, given that silanized interfaces become unstable in contact with moisture (1), RXU was the only resin cement capable of maintaining bond strength after aging, as it requires neither primer application nor silanization ( Table 1).

Saliva contamination has been shown to affect the resin bond to CAD/CAM ceramics and its durability (10). Accordingly, filtrations of the NaOClaq solution at the adhesive interfaces have lead to degradation of the resin matrix of both CEC (Figs. 5,6) and RXU (Fig. 7) polymeric materials (19,26), thus increasing the percentages of adhesive failures in most groups ( Table 3). Nonetheless, the results of this experiment provide only an indication of the possible performance of resin cements to zirconium and aluminum oxide-based ceramics and should be reasonably extrapolated to the clinical environment. Further in vitro long-term storage studies as well as controlled clinical trials are needed to redefine these findings (1,8). Within the limitations of this study, the following conclusions could be drawn: (a) resin-ceramic interfacial longevity depended on cement selection rather than on surface pre-treatments; (b) the MDP-containing (CEC) and the self-adhesive (RXU) resin cements provided adequate bond strength levels to alumina and zirconia CAD/CAM ceramics after challenging; (c) despite both cements being prone to degradation due to resin matrix dissolution, RXU luted to zirconia or untreated or sandblasted alumina showed the most stable interfaces; and (d) the conventional Bis-GMA resin cement (CAL) experimented spontaneous debonding in all tested groups regardless of the ceramic surface treatment.
